# Breast Cancer Inhibition by Biosynthesized Titanium Dioxide Nanoparticles Is Comparable to Free Doxorubicin but Appeared Safer in BALB/c Mice

**DOI:** 10.3390/ma14123155

**Published:** 2021-06-08

**Authors:** Haroon Iqbal, Anam Razzaq, Bushra Uzair, Noor Ul Ain, Shamaila Sajjad, Norah Ayidh Althobaiti, Aishah Eid Albalawi, Bouzid Menaa, Muhammad Haroon, Muslim Khan, Naveed Ullah Khan, Farid Menaa

**Affiliations:** 1 College of Pharmaceutical Science, Soochow University, Suzhou 215123, China; harooniqbal415@hotmail.com (H.I.); anamrazzaq.ajk@gmail.com (A.R.); noorulain22@yahoo.com (N.U.A.); naveedkhan1676@hotmail.com (N.U.K.); 2 Department of Biological Sciences, International Islamic University, Islamabad 44000, Pakistan; bushra.uzair@iiu.edu.pk (B.U.); shamaila.sajjad@iiu.edu.pk (S.S.); 3 Department of Biology, Faculty of Science and Humanities, Shaqra University, Al-Quwayiyah 11961, Saudi Arabia; nalthobaiti@su.edu.sa; 4 Department of Biology, Faculty of Sciences, University of Tabuk, Tabuk 47731, Saudi Arabia; ae.albalawi@ut.edu.sa; 5 Department of Oncology and Nanomedicine, California Innovations Corporation, San Diego, CA 92037, USA; bouzid.menaa@gmail.com; 6 Faculty of Pharmacy, Gomal University, Dera Ismail Khan 29050, Pakistan; haroon.pharma1717@gmail.com; 7 Department of Chemistry, Kohat University of Science and Technology, Kohat 26000, Pakistan; dr.muslim@kust.edu.pk

**Keywords:** *Zanthoxylum armatum* leaf extract, TiO_2_ nanoparticles, doxorubicin, cytotoxicity, apoptosis, reactive oxygen species, breast cancer, cardiotoxicity

## Abstract

Cancer remains a global health burden prompting affordable, target-oriented, and safe chemotherapeutic agents to reduce its incidence rate worldwide. In this study, a rapid, cost-effective, and green synthesis of titanium dioxide (TiO_2_) nanoparticles (NPs) has been carried out; Ex vivo and in vivo evaluation of their safety and anti-tumor efficacy compared to doxorubicin (DOX), a highly efficient breast anti-cancer agent but limited by severe cardiotoxicity in many patients. Thereby, TiO_2_ NPs were eco-friendly synthetized using aqueous leaf extract of the tropical medicinal shrub *Zanthoxylum armatum* as a reducing agent. Butanol was used as a unique template. TiO_2_ NPs were physically characterized by ultraviolet-visible (UV–Vis) spectroscopy, dynamic light scattering (DLS), transmission electron microscopy (TEM), scanning electron microscope (SEM), X-ray powder diffraction (XRD), and Fourier-transform infrared spectroscopy (FTIR) as routine state-of-the art techniques. The synthesized TiO_2_ NPs were then evaluated for their cytotoxicity (by MTT, FACS, and oxidative stress assays) in 4T1 breast tumor cells, and their hemocompatibility (by hemolysis assay). In vivo anti-tumor efficacy and safety of the TiO_2_ NPs were further assessed using subcutaneous 4T1 breast BALB/c mouse tumor model. The greenly prepared TiO_2_ NPs were small, spherical, and crystalline in nature. Interestingly, they were hemocompatible and elicited a strong DOX-like concentration-dependent cytotoxicity-induced apoptosis both ex vivo and in vivo (with a noticeable tumor volume reduction). The underlying molecular mechanism was, at least partially, mediated through reactive oxygen species (ROS) generation (lipid peroxidation). Unlike DOX (*P* < 0.05), it is important to mention that no cardiotoxicity or altered body weight were observed in both the TiO_2_ NPs-treated tumor-bearing mouse group and the PBS-treated mouse group (*P* > 0.05). Taken together, *Z. armatum*-derived TiO_2_ NPs are cost-effective, more efficient, and safer than DOX. The present findings shall prompt clinical trials using green TiO_2_ NPs, at least as a possible alternative modality to DOX for effective breast cancer therapy.

## 1. Introduction

Cancer remains a major death culprit worldwide and an obstinate threat to human health and life. In 2015, approximately 17.5 million new cancer cases and about 8.7 million cancer-related deaths occurred globally [1]. Comparatively, cancer cases increased at a rapid pace in 2018, with approximately 18.1 million new cancer cases and about 9.6 million deaths [2]. Approximately 22 million new cancer cases and about 13 million deaths are expected by 2030 [3]. The breast is one of the most susceptible organs to cancer (after lungs, liver, and stomach), and the incidence of breast cancer is relatively high (with eight out of ten women at risk of developing it), causing many deaths in industrialized countries among women aged between 35 and 55 years old [4,5].

Conventional treatment options include resection, chemotherapy, radiotherapy, hormonal therapy, or a combined therapy of these treatment regimen [6,7,8]. However, the complete ablation of tumor is exceedingly difficult due to the constrained region for the resection or surgery, drug-resistance development, and patients who encounter side-effects from the conventional therapy. Thereby, the survival rate at five years is still limited to about 20% [9]. Most recently, two immunotherapy drugs (PD-1/PD-L1 immune checkpoint inhibitors), namely atezolizumab and pembrolizumab, have been approved by the Food and drug Administration (FDA) but their use is restricted to patients with metastatic triple-negative breast cancer [10].

Hence, there is a continuous need to rationally design anti-cancer strategies for site-targeted drug(s) delivery while ensuring minimal toxic effects towards healthy tissues [11]. In this regard, nano-sized particles appeared as a budding strategy for cancer therapy with target-specificity, low toxicity, and speedy drug removal from the body and mature drug release at the tumor site [11,12,13,14]. In the hunt for new anticancer drugs, there is a significant progress in the fabrication and characterization of tailored metal oxide NPs for the treatment of cancers. Over the last two decades, oxide metallic nanostructures have been continuously designed, evaluated, and used in many applications [12,15,16]. Thereby, various metals (e.g., titanium, silver, zinc) were used to design metallic NPs via synthetic or natural methods, and strengthen the pharmaceutical and medical potential [12,16,17]. Indeed, metal NPs can activate the apoptotic pathway through ROS production, and subsequent anti-angiogenic, antiproliferative, and antitumor effects in vitro [16,18,19,20]. Among prominent NPs with demonstrations both in vitro and in vivo, TiO_2_ NPs show unique surface chemistry and morphologies (e.g., sizes and shapes), display a good biocompatibility, exert inherent biological activities (e.g., efficient antimicrobial and antitumoral properties) with weak side-effects and low eco-toxicity [21]. Previous studies reported that TiO_2_ NPs interfere with epidermal growth factor receptor (EGFR) signaling cascade, inducing ROS-mediated cytotoxicity and genotoxicity as central underlying molecular mechanisms that lead to cell apoptosis in malignant cells compared to neighboring physiological cells [20]. However, information about the relative therapeutic effect of TiO_2_ NPs for breast cancer compared to conventional therapies (e.g., DOX) is lacking. DOX is one of the most effective anticancer drugs to date, including for breast and ovarian carcinomas [22]. However, its clinical application is limited by its harmful side effects, the most significant of which is its cardiotoxicity, which can lead to cardiomyopathy and congestive heart failure [23]. Based on these observations, attempts have been made to develop novel drug delivery systems based on the encapsulation of DOX into TiO_2_ NPs with the goal to enhance DOX chemotherapeutic efficiency and reduce its side effects, but in an ex vivo setting only [24,25]. Recently, greenly fabricated silver-doped TiO_2_ NPs (i.e., Ag/TiO_2_) have been evaluated for their antimicrobial and anticancer activities but again in an in vitro setting only [26]. Additionally, it is worth noting that such doped TiO_2_ NPs nanocomposite are unlikely to be suitable as medicines in terms of safety. Indeed, a recent report demonstrated that Ag-doped TiO_2_ NPs induced toxicity in human liver cancer (HepG2) cells via oxidative stress, which increased with the increment of Ag level, suggesting that this was most likely due to the tuning of size and band gap of TiO_2_ NPs by Ag-doping [27]. Eventually, most of the published studies reported that TiO_2_ nanostructures prepared via different physical and chemical routes through bottom-up or top-down approaches (e.g., sol-gel, hydrothermal, solvothermal, hydrolysis, thermolysis, flame, and co-precipitation) [28,29]. These methods were sometimes combined with the principles of ‘green chemistry’ [15]. However, these synthesis approaches required relatively high temperature pressure, optimization of other parameters (e.g., pH, reaction time) or entail expensive and noxious chemicals, which make such TiO_2_ NPs unsuitable for their use as a safe theranostic modality [16,30]. Hence, the focus has shifted to the use of an eco-friendly, green, and cost-effective approach to synthesize, by combining principles of ‘green chemistry’ [15], or by metal bioreduction [16], various nanostructures with desired properties and less or no risk of hazardous chemicals [31]. Thereby, chemically ‘green’ syntheses of TiO_2_ nanostructures have been recently reported as valuable options to reduce eco-toxicity and lower the energy waste associated with chemicals. Indeed, TiO_2_ microtubes were synthetized via green sol-gel route using *Platanus acerifolia* seed fibers [32]. Additionally, green hydrothermal synthesis of TiO_2_ NPs was described using *Aloe barbadensis miller* (*Aloe vera*) gel and deionized water as starting materials [33]. Moreover, spinous hollow pure anatase TiO_2_ microspheres were obtained using a solvothermal green approach in which sunflower pollen acted as bio-templates [34]. Furthermore, spherical TiO_2_ NPs were produced via green co-precipitation method using *Phyllanthus emblica* (Amla) leaf extract and titanium tetraisopropoxide (TTIP) as a titanium source [35]. However, these green-chemical hybrid methods were all applied to produce TiO_2_ NPs as photocatalysts. The greenest and preferred choice for the NPs synthesis remains biological metal ions reduction to the corresponding metals from a natural source (e.g., plant extracts, microorganisms) which acts as a reducing agent/reductant to yield NPs with enhanced morphology (i.e., shape and size) and stability, in the absence of any chemicals/toxic solvents [16,31,36,37,38]. Such a method is cost-effective, allows the control of key parameters (e.g., morphologies, surface area, porosity) in the synthesis of doped or undoped TiO_2_ NPs [26]. Plants are considered the main factory for the green synthesis of metal oxide NPs, and until now, different plant species and plant parts (especially plant leaf extracts) have been used to study this. Unfortunately, studies using plant extracts as bioreductants to synthesize TiO_2_ NPs are limited. To date, studies reported the synthesis of TiO_2_ NPs from a range of (medicinal) plants (mostly aqueous leaf extracts) including *Acacia nilotica* (gum Arabic tree) [26], *Citrus limon* (lemon) [39], *A. vera* [40], *Allium cepa* (onion) [41], *Trigonella foenum-graecum* (fenugreek) [42], *Curcuma longa* (turmeric) [43], *Azadirachta indica* (neem) [44,45,46], *Euphorbia prostrata* (spurge spp.) [47], *Psidium guajava* (guava) [48], *Eclipta prostrata* (false daisy) [49], *Nyctanthes arbor-tristis* (night jasmine) [50], *Catharanthus roseus* (bright eyes) [51], but most of their applications have been focused on infectiology (as antimicrobials). Such efforts are largely justified (e.g., use of bio-precursors, no waste of chemicals, no toxicity, no energy waste associated with chemicals) for the development of a sustainable and scalable production of NPs.

To the best of our knowledge, this is a first report related to the biosynthesis of TiO_2_ NPs using *Z. armatum. Z. armatum* (also called prickly ash, and commonly known as “Timer”) belongs to the family *Rutaceae* (genus Fagara). It is a spiny and deciduous shrub endemic to Pakistan and China. Various parts of this tall aromatic plant are used in indigenous systems of medicine because they exert antimicrobial, hepatoprotective, anti-inflammatory, and antioxidant activities [52].

Owing to the limited information on the usage of plant extracts for synthesizing TiO_2_ NPs and the rarity of data highlighting in vivo chemotherapeutic effects of undoped and unloaded NPs on breast cancer, our present work aimed to, in an ecofriendly manner, synthetize TiO_2_ NPs using *Z. armatum* aqueous leaf extract as an original reducing agent through a fast, simple, cost-effective, and easy scheme. The prepared TiO_2_ NPs were characterized employing a wide-range of routine state-of the-art techniques (e.g., SEM, TEM, XRD, FTIR, DLS, UV-Vis spectrophotometry). Since most of the studies related to TiO_2_ NPs have been centered around photocatalysis, antimicrobial potential in vitro, and anti-cancer activity ex vivo, we undertook their exploration as potential anti-cancer chemotherapeutic not only ex vivo using 4T1 breast cancer cell line but also in vivo using a subcutaneous 4T1 breast mouse tumor model. Their efficiency and side effects were compared to DOX and phosphate-buffered saline (PBS, 1X, pH 7.4), used as positive control (PC) and negative control (NC), respectively.

## 2. Material and Methods

### 2.1. Plant Collection and Preparation of Leaf Extracts

*Z. armentum* was collected from Rawalakot, Azad Kashmir, Pakistan, during the spring season 2019 and confirmed by an expert botanist, Faculty of Pharmacy, Gomal University D. I. Khan, KPK, Pakistan. The plant extracts were made using dried crushed leaves.

A total of 50 g of the sieved leaf powder was slowly added to 500 mL of sterile deionized water (DH_2_O), subsequently boiled for 10 min and kept in the dark for two days at 30 °C. Thereafter, the plant blend was filtered, and the resulting aqueous extract was used for the preparation of TiO_2_ NPs.

### 2.2. Green Synthesis of TiO_2_ NPs

TiO_2_ NPs were freshly synthesized by using titanium tetra butoxide (TBT, Ti(OBu)_4_) as a precursor, butanol as a template, and plant extract as a reducing agent. A total of 10 mL of TBT was added dropwise to 100 mL aqueous extract of plant and 25 mL of butanol. This solution was heated at 65 °C and kept on shaker incubator for two hours. After this time duration, the color of the reaction mixture changed from normal bluish watercolor to brown, indicating the formation of TiO_2_ NPs. These NPs were collected by centrifugation (10,000 rpm, 15 min), washed with double distilled water (ddH_2_O), and kept in a drying oven at 60 °C for 24 h. The resulting powdered NPs were additionally subjected to calcination process inside a furnace for 2 h at 500 °C.

### 2.3. Physicochemical Characterizations of the Green Synthesized TiO_2_ NPs

The synthesis of the TiO_2_ NPs from *Z. armentum* leaf extract was confirmed by UV-Vis spectroscopy (Shimadzu UV-2600 Spectrometer, Kyoto, Japan) using the wavelength range of 800–200 nm [40].

The crystalline structure of the synthesized NPs was examined by XRD (Bruker D8 Advance, Billerica, MA, USA). The Bruker D8 Advance apparatus has a theta:theta geometry (often called Bragg-Brentano or focusing geometry) with a copper sealed tube ray source producing Cu kα irradiation (technically kα1 and kα2 with kβ being removed by the primary optic) at a wavelength of 1.5406 Å from a generator operating at 40 kV and 40 mA. Data collections used detector scans at a grazing incidence angle ranging from 10° to 80°. The samples were then analyzed for their average crystallite size [53,54]. 

The average hydrodynamic particle size (PS)/particle size distribution (PSD) of TiO_2_ NPs was determined by DLS (Malvern Zetasizer Nano ZS90, Malvern, UK) using distilled water (dH_2_0) as solvent at 90° scattering angle with 30 s equilibrium time between 3 cycles.

The average core size and surface morphology of TiO_2_ nanoparticles was obtained by TEM (Hitachi H-600, Kyoto, Japan) at 200 kV, and by SEM (Tescan Mira3 FEG-SEM, Brno, Czech Republic) at the accelerating voltage of 10 kV, respectively.

FTIR spectrometer (Thermo/Nicolet MAGNA-IR 560, Champaign, IL, USA) was employed at 500–4000 cm^−1^ to qualitatively determine the IR-active functional groups or bonds in the TiO_2_ NPs. Briefly, 0.02 g of TiO_2_ NPs were grounded with 0.2 g of potassium bromide (KBr) and then pressed into pellet form using desktop Powder Presser/dry pressing machine EQ-YLJ-24T (MTI, Seoul, Korea).

### 2.4. Cellular Uptake of the Green Synthesized TiO_2_ NPs

The cellular uptake of the prepared TiO_2_ NPs was evaluated in murine 4T1 mammary carcinoma cells (ATCC (Manassas, VA, USA) following a previous method with minor modifications [55]. Briefly, 4T1 cells were seeded in 12-well plates at density of 1 × 10^5^ cells/well in RPMI-1640 supplemented with 10% fetal bovine serum (FBS)) (Merk, Darmstadt, Germany). After 24 h incubation at 37 °C, the cells were treated using a range of concentrations (0.5, 1, 2, 5, or 10 µg/mL) of TiO_2_ NPs, to evaluate the cellular uptake of Ti in a concentration-dependent manner. The plates were further incubated for 2, 4, 6 or 12 h. Untreated cells (0 µg/mL TiO_2_ NPs) were used as control. Subsequently, the cells were washed five times with PBS (1X, pH 7.4), trypsinized with 0.5 mM Trypsin/EDTA to detach the cells from the bottom of the plates, collected by centrifugation, and dispersed in 2 mL PBS (1X, pH 7.4). Eventually, cells were accurately counted using a hemocytometer, ruptured using a mixture of perchloric acid and aqua-regia at 280 °C to extract Titanium (Ti), whose concentration was determined by inductively coupled plasma optical emission spectroscopy (ICP-OES).

### 2.5. Ex Vivo Cytotoxicity of the Green Synthesized TiO_2_ NPs

Murine 4T1 cells were seeded in plates containing RPMI-1640 supplemented with 10% FBS and incubated at 37 °C for 24 h. Cells were harvested with 0.5 mM Trypsin/EDTA when cell confluency reached about 90%. The in vitro cytotoxic activity of TiO_2_ NPs was then evaluated using MTT assay as previously reported [56]. Briefly, 4T1 cells were seeded in 96-well plates at the density of 5 × 10^3^ cells per well and allowed to grow for 24 h at 37 °C. After 24 h incubation, 100 μL of cell culture medium containing a given concentration (range: 0–32 µg/mL) of either TiO_2_ NPs or DOX, used as PC, were added to each well. Untreated cells were used as NC. After incubation overnight, each well was washed with PBS (1X, pH 7.4) thrice before 20 µL MTT reagent (5 mg/mL) was added followed by the addition of 100 μL of fresh cell culture medium. After 4 h incubation, the cell medium was aspirated and 150 μL of pure Dimethylsulfoxide (DMSO, 100%) was added to dissolve the formazan. Eventually, the absorbance of formazan, which provides a direct estimate of the number of living cells, was measured at 492 nm using the easy-to-use multimode plate reader infinite 200 PRO (TECAN). To ensure the data reliability, the experiment was conducted in triplicate. The normalized percentage (%) of cell viability was calculated as follows:

(1) $$\mathrm{Cell}\;\mathrm{viability}\;\left(\%\right)=\frac{\mathrm{absorbance}\;\mathrm{of}\;\mathrm{sample}}{\mathrm{absorbance}\;\mathrm{of}\;\mathrm{control}}\times 100$$

### 2.6. Evaluation of the Green TiO_2_ NPs-Induced Cell Apoptosis by Flow Cytometry

For apoptosis study, murine 4T1 cells (3 × 10^5^ cells/well) were cultured in 6-well plates and incubated for growth for 24 h in RPMI-1640 supplemented with 10% FBS. Subsequently, the cells were treated for 24 h with TiO_2_ NPs (5 µg/mL). PBS (1X, pH 7.4), and DOX (5 µg/mL) were used as NC and PC, respectively. The cells were then washed, trypsinized, collected, and dispersed in 500 µL PBS (1X, pH 7.4). Afterwards, the resuspended cells were stained with FITC-Annexin V, following the manufacturer’s instructions of the Apoptosis Detection Kit I (BD Bio Sciences, San Jose, CA, USA). Eventually, the rate cell apoptosis was evaluated by FACStar-Plus flow cytometry (Becton Dickinson, Franklin Lakes, NJ, USA).

### 2.7. Lipid Peroxidation

Lipid peroxidation (LPO) induced by TiO_2_ NPs were evaluated by thiobarbituric acid reactive substance (TBARS) assay [26]. Briefly, 4T1 cells exposed to 5, 10, or 20 µg TiO_2_ NPs were centrifuged at 5000 rpm at 4 °C, and the supernatant was collected. Untreated cells were used as control. Then, 2 mL of TBARS was added to 1 mL of each supernatant, and the mixture was eventually heated to 95 °C for 60 min, according to the manufacturer’s instructions. Subsequently, the samples were cooled using an ice bath and centrifuged. The absorbance of each supernatant (upper layer) was recorded at 532 nm using the multimode plate reader infinite 200 PRO (TECAN, Grodig/Salzburg, Austria). The normalized percentage (%) of LPO was calculated as follows:

(2) $$\mathrm{LPO}\;\left(\%\right)=\frac{\mathrm{absorbance}\;\mathrm{of}\;\mathrm{sample}}{\mathrm{absorbance}\;\mathrm{of}\;\mathrm{control}}\times 100$$

### 2.8. Hemocompatibility of the Green TiO_2_ NPs by Hemolysis Assay

The hemocompatibility of TiO_2_ NPs was evaluated *in vitro* using red blood cells (RBCs) of BALB/c mice, considering that blood is the gateway for all NPs to reach their target tissues or organs. Briefly, 2 mL of TiO_2_ NPs used at different concentrations (0.1–1 mg/mL) were added into 2 mL (2% *v/v*) of fresh murine RBCs in PBS (1X, pH 7.4). The solutions were then incubated in a water bath at 37 °C for 2 h. Subsequently, the solutions were centrifuged at 4 °C for 15 min at 1500 rpm, before the absorbance of each supernatant (upper layer) was recorded at 540 nm using the multimode plate reader infinite 200 PRO (TECAN). RBCs treated with ddH_2_O or PBS (1X, pH 7.4) served as PC and NC, respectively. Hemolysis rate (HR) was then calculated as follows:

(3) $$\mathrm{HR}\;\left(\%\right)=\frac{X\;\mathrm{sample}-X\;\mathrm{negative}}{X\;\mathrm{positive}-X\;\mathrm{negative}}\times 100$$

where X is the absorbance of the UV spectrum.

### 2.9. In Vivo Evaluation of the Green TiO_2_ NPs as Breast Cancer Chemotherapeutic Agent

The subcutaneous 4T1 tumor model was first established by injecting 1 × 10^6^ 4T1 cells suspended in 50 μL PBS (1X, pH 7.4) into the right lower flank of 6/8-week-old BALB/c mice (*N* = 15). When the tumors achieved an average volume of 70–80 mm^3^ (5 days post tumor induction), they were randomly divided into 3 treatment groups (*n* = 4/group): Group (1) = PBS (1X, pH 7.4; 20 mL/kg/day) used as NC; Group (2) = free DOX (5 mg/kg/day) used as PC; and Group (3) TiO_2_ NPs (5 mg/kg/day) used as Test. PBS, DOX or TiO_2_ NPs were then injected intravenously (IV) at day 5, 8 and 11 post-tumor induction, for a total of three doses.

To evaluate the therapeutic response, the tumor size/growth and body weight of each group of mice were measured every two days (3× weekly) by using digital Vernier calipers and analytical weighing balance, respectively. Eventually, the tumor volume was calculated by the formula:

(4) $$\mathrm{Tumor}\;\mathrm{Volume}\;=\;\mathrm{Length}\;\times \frac{{\mathrm{Width}}^{2}}{2}$$

Twenty-one days later, at the end of experiment, all the mice were simultaneously sacrificed by cervical dislocation [57]. Tumors from each group were subsequently excised, weighed, and photographed. To monitor histopathological changes, the harvested breast tumor specimens from all groups (NC, PC, and Test) were fixed in 10% neutral buffered formalin, embedded in paraffin blocks, and cut into 4-μm-thick serial sections. The same was done with other major organs (heart, liver, kidney, lung, and spleen). Organ sections were processed and stained with hematoxylin and Eosin (H & E) staining, according to routine protocols [58].

### 2.10. Ethics Statement

The animal experiments using mice were performed in strict accordance with the Regulations for the Administration of Affairs Concerning Experimental Animals (1988.11.1), and all efforts were made to minimize suffering. All procedures concerning animal usage were reviewed and approved (on 19 December 2019) by the Institutional Animal Care and Use Committee of Kohat University, KPK, Pakistan, for the use of laboratory animals (Permit Number: 2019-89).

### 2.11. Statistical Analysis

To ensure accuracy of the data and their reproducibility, all experiments were triplicated independently. The data were expressed as mean ± standard deviation (SD). Statistical analyses of tumor size and weight in the animal studies were conducted using the Student’s *t*-test and Origin Pro8 software [57]. * *P*-values < 0.05 were considered significant while ^#^ *P*-values > 0.05 were considered insignificant.

## 3. Results and Discussion

### 3.1. Eco-Friendly TiO_2_ NPs Were Successfully Synthesized

**Figure 1:**
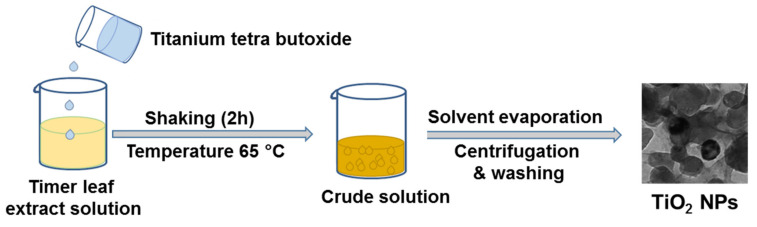
Biosynthesis of TiO_2_ NPs. Here, leaf extract of *Z. armatum* and butanol were applied as reducing agent and templating agent, respectively.

The basic idea is to use a simple, fast, cost-effective, and green process to hydrolyse an inorganic material precursor in the form of droplets. For this purpose, we used a specific optimized ratio of a plant extract and alcohol. The synthesis scheme of TiO_2_ NPs from leaf extract of *Z. armatum* is depicted in Figure 1.

Briefly, when butanol was added in *Z. armatum* aqueous leaf extract, the mixture led to an emulsion-like environment due to the larger carbon chain length of butanol. *Z. armatum* aqueous leaf extract worked as a unique reducing agent, while butanol worked as a templating agent. The larger alkoxy groups of butanol favored its insolubility in plant extract, providing a polymeric templating effect. Interestingly, these insoluble alcohol droplets are deformable and can easily be removed by evaporation on low heating. In a previous study, such alcohol droplets behaved as templates for the growth of mesoporous TiO_2_ NPs by a simple sol-gel technique [18].

Previous studies reported the synthesis of TiO_2_ NPs using different plant species such as *P. guajava* [48], *C. longa* [43], or *C. limon* [39], but most of them were intended for their evaluation as potential antimicrobials. The usefulness of this green approach for the production of macroporous materials with tunable sizes have advantages (e.g., cost-effectiveness, rapidity, easiness, stability, and high yield of NPs, reliability/reproducibility) over other existing routes (physicochemical ones) [16,29].

Then, the phytogenic TiO_2_ NPs were characterized by various physical techniques used routinely for such purpose (i.e., UV-Vis, DLS, TEM, SEM, XRD, FTIR).

**Figure 2:**
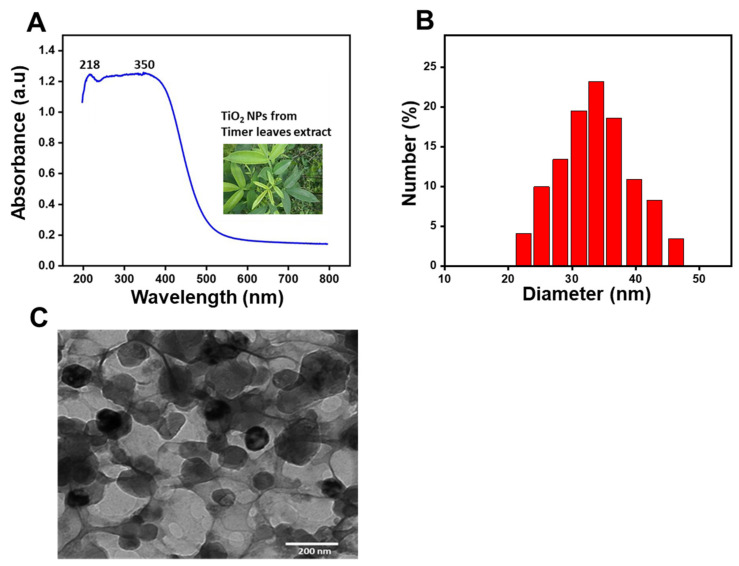
(**A**) UV–Visible spectrum of synthesized TiO_2_ NPs using timer leaf extracts; (**B**) Histogram showing the size distribution of the green TiO_2_ NPs assessed by DLS; (**C**) TEM image of the green TiO_2_ NPs.

Thereby, the synthesis of TiO_2_ NPs was primarily confirmed by UV-Vis spectroscopy. As depicted in Figure 2A, strong peaks of absorbance appeared at 218 nm and 350 nm, confirming the fabrication of TiO_2_ NPs. Indeed, Rajkumari et al. previously described that TiO_2_ NPs exhibited characteristic peaks of absorbance at 217.60 and 350.47 nm [40].

Then, the hydrodynamic PS and polydispersity index (PDI) of the TiO_2_ NPs were measured by DLS. As shown in Figure 2B, TiO_2_ NPs exhibited a suitable hydrodynamic PS of 37.33 ± 2 nm with a PDI of 0.27.

Further, PS of the green TiO_2_ NPs was assessed by TEM analysis. As shown in Figure 2C, these TiO_2_ NPs exhibited an average particle core size of about 16.2 ± 2 nm with a narrow size distribution and a spherical geometry. Our findings are in line with previous studies which reported spherically shaped and small TiO_2_ NPs synthetized from leaf extracts of the *A. indica* with a size that ranged from 15 to 50 nm [46], or *A. vera* with an average size of 20 nm [40]. TiO_2_ NPs with such a small size range are suitable for escaping rapid renal excretion, as well as avoiding components of the reticular endothelial system (RES), thus (i) facilitating potentially passive targeting of drugs to tumors via the enhanced permeation and retention (EPR) effect, and (ii) increasing drug accumulation in tumor cells after endocytosis [59].

**Figure 3:**
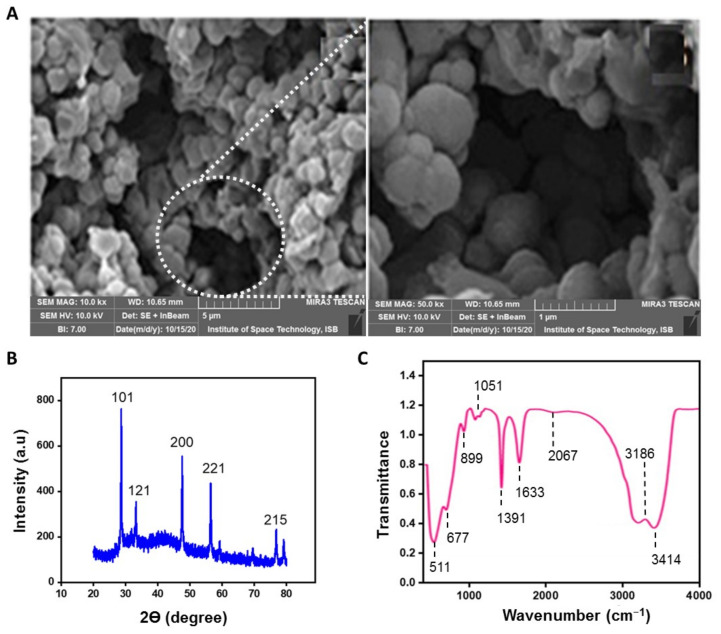
(**A**) SEM images of the biosynthesized TiO_2_ NPs (**Left**: scale bar 5 µm, magnification 10×; **Right**: scale bar 1 µm, magnification of the selected area 50×); (**B**) XRD of the biosynthesized TiO_2_ NPs; (**C**) FTIR spectrum of the biosynthesized TiO_2_ NPs.

Additionally, the surface morphology of TiO_2_ NPs was assessed by SEM. TiO_2_ NPs, prepared via a simple template-free route, displayed an average pore size of 3 ± 0.55 nm with homogeneous distribution as shown in Figure 3A. The micrographs showed the incredible effect of the butanol and plant extract on the morphology of TiO_2_ NPs. The high-resolution SEM images also revealed that TiO_2_ NPs are composed of smaller sized spherical particles with a range of 15 to 30 nm. Hence, the findings are consistent with TEM analyses, and other studies performed with *A. vera* (average PS of 20 nm) [40] or with *A. indica* (range PS of 25–87 nm) [46]. The mesoporosity among the titania structure arises due to the inter-particle and intra-particle porosity [28].

Besides, XRD analysis was carried out to provide detailed and rapid information about the crystallinity and phase purity of the biosynthesized TiO_2_ NPs. The Figure 3B revealed that TiO_2_ NPs display five distinctive and sharp diffraction peaks with 2 theta (θ) values located at the (215), (200), (101), (121) and (221) crystal planes (a.u). As confirmed by Joint Committee on Powder Diffraction Standards library (JCPDS Card no. 21-1272), the observed patterns at the (215), (200) and (101) crystal planes (a.u) correspond to the anatase phase, whereas the other characteristic peaks sited at the (121) and (221) crystal planes (a.u) correspond to the brookite phase and the rutile structure, respectively. Henceforth, the biosynthesized TiO_2_ NPs represent a combination of brookite, rutile, and anatase phases. Altogether, the XRD pattern revealed the crystalline nature of TiO_2_ NPs. The mean crystal size of the resultant mesoporous-TiO_2_ NPs was 5.41 ± 1 nm, as calculated using Scherrer equation [54]. Our findings are highly consistent with previous data on TiO_2_ NPs synthesized from *C. longa* extract [28,43]. Eventually, the biosynthesis of TiO_2_ NPs was validated by FTIR spectroscopy, which collected high-spectral-resolution data over the wide spectral range of 500–4000 cm^−1^, to identify active functional groups (Figure 3C). The prominent peak observed at 3414 cm^−1^ is assigned to O-H stretching vibrations of alcohols and phenolic compounds (e.g., flavonoids) in the leaf extract of *Z. armatum* [52,60]. Similar observations have been observed for TiO_2_ NPs synthesized from *A. indica* or *E. prostrata*, with a peak at 3421 cm^−1^ or at 3417 cm^−1^, respectively [44,49]. The peak at 3186 cm^−1^ can be attributed to wide O–H stretching vibrations. There is no peak at 2900 cm^−1^ regarding C–H stretching band, which means that all organic compounds were removed from the samples after calcinations [61]. The absorption band at 2067 cm^−1^ corresponds to the vibrations of C = C group [44]. The peak centered at 1633 cm^−1^ is characteristic of *δ*-H_2_O bending (surface-adsorbed water) and vibration of hydroxyl groups [40,61]. The peak at 1391 cm^−1^ may be attributed to C = C groups of aromatic rings [46]. The absorption band at 1051 cm^−1^ denotes the C = O vibrations of carboxylic acids, and alcohols. The peak observed at 677 cm^−1^ corresponds to Ti-O-Ti stretching vibration of the anatase TiO_2_ NPs [44]. The prominent absorption peaks observed between 511 cm^−1^ and 899 cm^−1^ resembles the specific vibrational norms of the anatase TiO_2_ [61]. These overall findings are consistent with previous studies reporting (i) the synthesis of TiO_2_ NPs from plants source such as *A. vera*, *A. indica*, and *C. longa* extracts, and (ii) the existence of flavonoids, terpenoids and proteins that more likely acted as reducing and capping agents in the process of NPs synthesis and stabilization [40,43,44,46]. 

### 3.2. The Uptake of TiO_2_ NPs by Breast Cancer Cells Is Time- and Dose-Dependent

**Figure 4:**
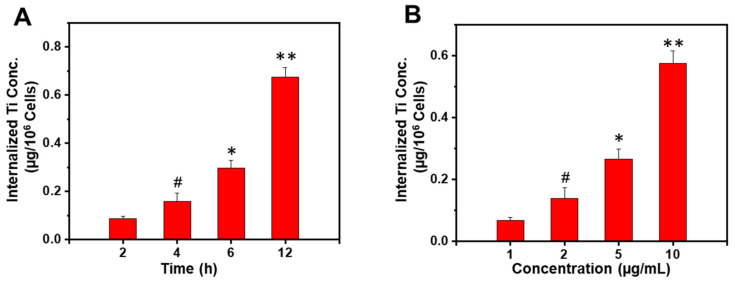
Cellular uptake of TiO_2_ NPs by 4T1. (**A**) Time-dependent (5 µg/mL TiO_2_ concentration); (**B**) Concentration-dependent (6 h incubation). Data are expressed as a mean ± SD, (** *P* < 0.01, * *P* < 0.05, # *P* > 0.05, compared to the previous time or concentration used).

In a further step, we evaluated the capacity of TiO_2_ NPs to be interiorized by 4T1 breast tumor cells. As revealed in Figure 4, TiO_2_ NPs exhibited both time- and concentration-dependent cellular uptakes, reaching their maximal uptake at 6 h when 10 µg/mL of TiO_2_ NPs (*P* < 0.01) were used or at 12 h with 5 µg/mL TiO_2_ NPs (*P* < 0.01). Such an effect is known to be highly beneficial for the small TiO_2_ NPs to induce ROS-mediated cytotoxicity and genotoxicity in cancer cells, leading to cell death through alterations in the phosphorylation status of proteins downstream of the epidermal growth factor receptor (EGFR) signaling cascade (e.g., Akt, Erk) [20].

### 3.3. Z. Armatum-Derived TiO_2_ NPs Exert DOX-Like Cytotoxicity on Breast Cancer Cells

To check the potential cytotoxic effect of the greenly synthetized TiO_2_ NPs, MTT assay was carried out in 4T1 mammary carcinoma cells using TiO_2_ NPs at the concentration range (0–32 μg/mL) and DOX as PC. Untreated cells were used as NC. DOX is one of the most important anticancer agents used in treating breast cancer [62].

**Figure 5:**
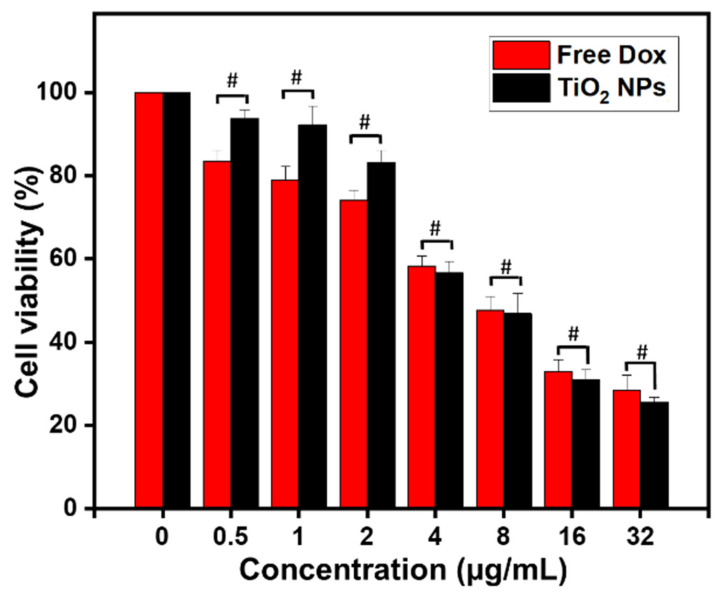
Relative viability of 4T1 cells after treatment for 24 h (at 37 °C) with the indicated concentration of biosynthesized TiO_2_ NPs. Free DOX at the same indicated concentrations was used as PC. Data are expressed as mean ± SD. # *P* > 0.05.

As shown in Figure 5, free DOX showed IC_50_ (half maximal inhibitory concentration) at 5.29 μg/mL. Interestingly, TiO_2_ NPs showed IC_50_ at 4.11 μg/mL (*P* > 0.05). Thus, it can be concluded that the biosynthesized TiO_2_ NPs exhibit comparable anticancer efficacy and toxicity in 4T1 breast cancer cells compared to that of DOX. Besides, our data showed that 4T1 breast cancer cells might be quite resistant to DOX and TiO_2_ NPs when their respective effects are compared to that observed in human SMMC-7721 hepatocarcinoma cells. Indeed, IC_50_ value was as low as 0.32 μg/mL when SMMC-7721 cells were treated with DOX while about 95% of the cells were alive when treated by TiO_2_ NPs at 10 μg/mL [24].

To the best of our knowledge, this is the first study reporting cytotoxicity effects of unloaded/undoped TiO_2_ NPs in 4T1 mammary carcinoma cells. Although Chen et al. concluded that such NPs lack cytotoxicity in hepatocarcinoma cells [24], Rao et al., reported an anti-cancer activity of Ag-doped TiO_2_ NPs against MCF-7 human breast carcinoma cell line, and stated that cytotoxicity was mainly mediated by ROS generation and oxidative stress [26]. Our observations fit with a new paradigm shift and allow us to postulate that the anti-cancer effect of unloaded/undoped TiO_2_ NPs could be due to the type and conditions of NPs synthesis, NPs characteristics, and cell line/type. In further steps, we thus decided to characterize the cell death (e.g., apoptosis, necrosis), and define the primary molecular mechanism by which such specific greenly synthesized TiO_2_ NPs induce tumor cell death.

### 3.4. Z. Armatum-Derived TiO_2_ NPs Induce Apoptosis in 4T1 Breast Cancer Cells

Although it is well-assumed that TiO_2_ NPs can generate ROS in cells, including in human breast cancer cells [20], no previous report has riveted on whether *Z. armatum*-derived TiO_2_ NPs can promote cell death in murine 4T1 breast cancer cells.

To answer this hypothesis, we applied a double-staining (PI/Annexin V) method and calculated the number of live cells by a statistical gating approach using FACS to evaluate the TiO_2_ NPs-induced cell death in 4T1 cells [63]. The Annexin V provides a sensitive method for detecting cellular apoptosis while PI is used to detect necrotic or late apoptosis characterized by the loss of membrane integrity [64].

**Figure 6:**
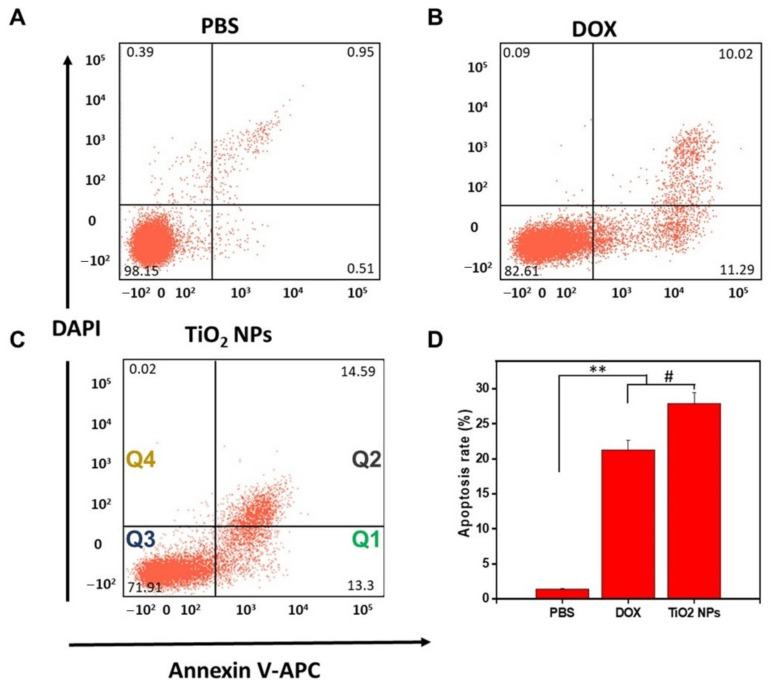
FACS evaluation of Annexin V/PI double stained 4T1 breast cancer cells after treatment for 24 h. (**A**) with PBS (1X, pH 7.4) used as NC, (**B**) with DOX (10 µg/mL) used as PC, (**C**) with TiO_2_ nanoparticles (10 µg/mL), (**D**) relative apoptotic rate (%) of treated 4T1 cells (** *P* < 0.05, # *P* > 0.05). Q1: PI/FITC −/+ apoptotic cells; Q2: PI/FITC +/+ late apoptotic cells; Q3: PI/FITC −/− viable cells; Q4: PI/FITC +/− necrotic cells.

The level of cell death was evaluated in TiO_2_ NPs-treated 4T1 cells comparatively to DOX-treated 4T1 cells (PC) and PBS-treated 4T1 cells (NC), as shown in Figure 6. TiO_2_ NPs and DOX were used at the same concentration (10 µg/mL). The data generated by flow cytometry are plotted in two-dimensional dot plots in which PI is represented versus Annexin V-FITC. These plots can be divided in four regions corresponding to: (1) apoptotic cells which are PI negative and Annexin positive (PI/FITC −/+; Q1); (2) late apoptotic cells which are PI and Annexin positive (PI/FITC +/+; Q2); (3) viable cells which are negative to both probes (PI/FITC −/−; Q3); (4) necrotic cells which are PI positive and Annexin negative (PI/FITC +/−; Q4).

Negligible necrotic cells (<0.39%) were observed after PBS treatment (Figure 6A). The rate of apoptosis in 4T1 cells treated with TiO_2_ NPs (27.89 ± 3.2%) (Figure 6C) was found to be slightly higher (*P* > 0.05) compared to that of 4T1 cells treated with DOX (21.31 ± 2.4%) (Figure 6B). However, this average rate of apoptosis, in either TiO_2_ NPs-treated 4T1 cells or DOX-treated 4T1 cells, was drastically (*P* < 0.01) higher compared to that of 4T1 cells-treated with PBS (1.46 ± 0.8%).

Taken together, our results show that TiO_2_ NPs induce apoptosis in 4T1 breast cancer cells in a similar fashion compared to that of DOX, strengthening our data obtained from MTT cytotoxicity assays.

### 3.5. The Green TiO_2_-Induced Cell Apoptosis Is Mediated by Lipid Peroxidation

LPO refers to the degradation of lipid in cell-membrane under oxidative stress mediated by ROS generation. It is well-documented that lipid hydroperoxides, oxidative lipid degradation, and ROS generation can bestow in signal transduction pathways cell growth, differentiation, maturation, and cell death (apoptosis) [26].

**Figure 7:**
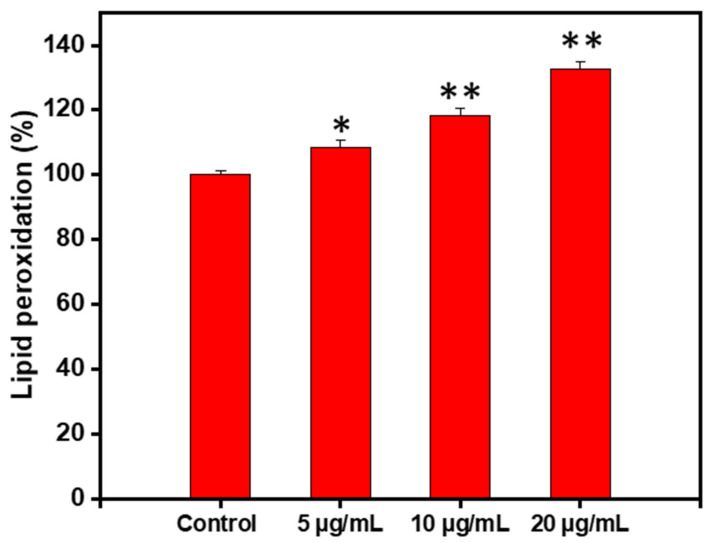
Percent change in lipid peroxidation in 4T1 cells treated for 1 h with TiO_2_ NPs at the indicated concentration. Data are expressed as mean ± SD (* *P* < 0.05, ** *P* < 0.01 compared to untreated cells, used as NC).

Consequently, the effect of TiO_2_ NPs on LPO in 4T1 cells was evaluated. Interestingly, LPO significantly increased (*P* < 0.05) with increased concentration of TiO_2_ NPs as depicted in Figure 7. Thus, the TiO_2_-induced apoptosis in 4T1 cells can be, at least in part, ascribed to lipid peroxidation.

### 3.6. The PhytogenicTiO_2_ NPs Are Hemocompatible

Eventually, one of the important aspects for the in vivo application of nanomaterials is the hemocompatibility, as the injected nanomaterials interact firstly with RBCs before the immune cells [37,65]. Therefore, the hemolysis assay is considered an important feature for preclinical study.

**Figure 8:**
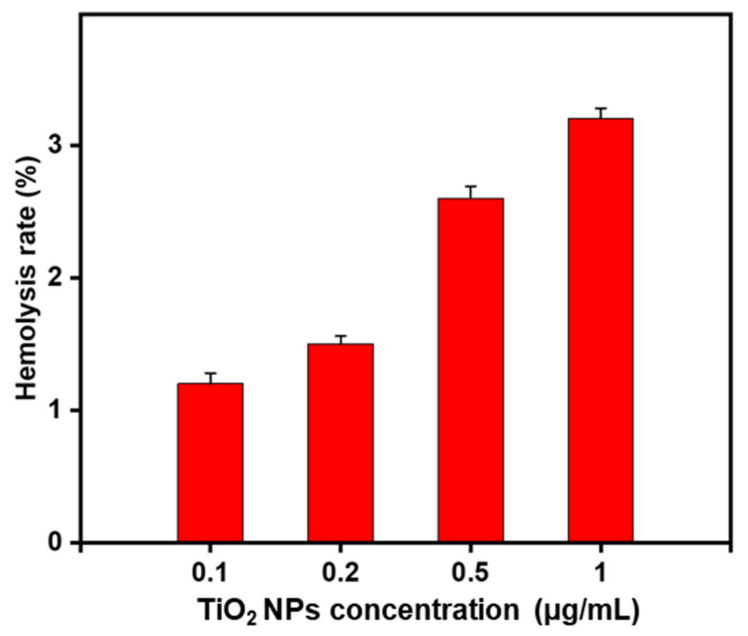
Hemolysis rate (%) of RBCs (2% *v/v*) treated for 2 h (at 37 °C) with the biosynthesized TiO_2_ NPs at various indicated concentrations. ddH20 was used as PC and PBS (1X, pH 7.4) was used as NC.

Hence, we checked the potential hemolytic activity of TiO_2_ NPs. The hemolysis assay consisted of using a concentration range of 0.1–1 mg/mL of TiO_2_ NPs, each of which concentration was added to a fresh RBC solution (2% *v/v*). The hemolysis rate (HR), that reflects the % of RBCs affected by the NPs, was calculated (Figure 8).

Interestingly, the effect of TiO_2_ NPs on HR was dose-dependent but was still extremely low (<4%) at the remarkably high concentration of 1 mg/mL (equivalent to the dose of 2 mg).

According to the criterion in ASTM E2 S24-08 and ISO/TR 7406 international standards, compound-induced hemolysis >5% is considered as toxic [37]. Thus, our data demonstrated outstanding hemocompatibility of the phytosynthesized TiO_2_ NPs, suggesting that these NPs could be safely translated for in vivo assays, when using IV administration route.

### 3.7. Phytogenic TiO_2_ NPs Cause DOX-Like Tumor Growth Inhibition

The TiO_2_ NPs-induced cytotoxicity previously observed in 4T1 cells indicated that TiO_2_ NPs might reduce the in vivo tumor growth as well.

To check this hypothesis, potential in vivo inhibitory effects of TiO_2_ NPs were evaluated by using a murine model of subcutaneous 4T1 mammary carcinoma. This model was created by injecting 1 × 10^6^ 4T1 cells suspended in 50 μL PBS (1X, pH 7.4) into the right lower flank of 6/8-week-old BALB/c mice (*N* = 15). Three experimental groups (Group I: PBS (1X, pH 7.4; 20 mL/kg/day); Group II: free DOX (5 mg/kg/day), and Group III: TiO_2_ NPs (5 mg/kg/day)), with five mice per group, were enrolled in this study.

**Figure 9:**
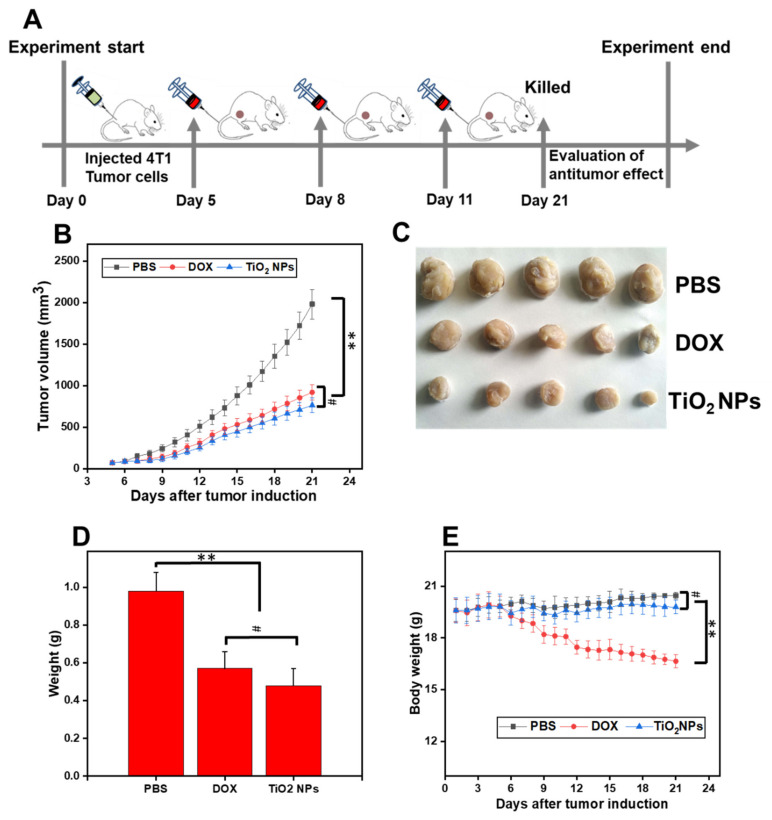
From tumor induction in mice to TiO_2_-induced tumor regression. (**A**) Creation of a BALB/c Model of 4T1 Mammary Carcinoma and evaluation of the time-course effects after injection, via tail vein injection at day 5, 8, and 11 post-tumor induction, of either PBS (1X, pH 7.4; 20 mL/kg/day), free DOX (5 mg/kg/day), or TiO_2_ NPs (5 mg/kg/day). At the end of the study (day 21), the mice were simultaneously sacrificed; (**B**) Graphical evolution of the tumor growth in treated mice (from day 5 post-tumor induction to day 21); (**C**) Photograph of excised breast tumors from the three mice groups (*n* = 5 per group), at day 21 post-tumor induction; (**D**) Weight of excised tumor after treatment with the indicated regimen; (**E**) Body weight of tumor-bearing mice during the course study. ** *P* < 0.01, ^#^ *P* > 0.05.

Five days post-tumor induction, the tumor volume reached a similar palpable stage (approximately 70–80 mm^3^, *P* > 0.05) in all mice (*N* = 15, *n* = 5 mice/group). At days 5, 8, and 11 post-tumor induction, PBS (20 mL/kg/day), free DOX (5 mg/kg/day) or TiO_2_ NPs (5 mg/kg/day) were injected via tail vein injection into each mouse of each specific group (Figure 9A). Then, the therapeutic response (i.e., by means of tumor size/growth, and body weight) of each mouse (from the different groups) was measured daily from day 5 post- tumor Induction until the day 21, the day of the simultaneous sacrifice (Figure 9A). Induced breast tumors from each mouse group were eventually excised, weighed, and photographed.

As shown in Figure 9B, the growth profile of 4T1 primary tumors in mice injected with TiO_2_ NPs began to revert from the seventh day post-tumor induction until the day of sacrifice. Interestingly, during the full-time course, the effect of TiO_2_ NPs on primary tumor volume was comparable to that of the free DOX (*P* > 0.05) but was significantly lower (up to 3-fold reduction, *P* < 0.05) compared to that of PBS (Figure 9B,C). In agreement with these findings, no significant changes (*P* > 0.05) were noticed in the weight of breast tumors excised at day 21 post-tumor induction when the TiO_2_ NPs-treated mice group was compared to that of the free DOX-treated mice group; however, the breast tumor weight of these groups was found to be significantly lower (*P* < 0.01) than the weight of breast tumors in the PBS-treated group (Figure 9C,D). Importantly, there was a significant difference between the body weight of mice from the TiO_2_ NPs-treated group compared to that of the free DOX-treated group (*P <* 0.01); However, insignificant (*P >* 0.05) difference was observed in the body weight of mice treated with TiO_2_ NPs compared to that of mice treated with PBS (Figure 9E).

Taken together, these pioneered data strongly indicate that undoped and *Z. armatum*-derived TiO_2_ NPs exert a comparable potent anti-breast tumor activity to free DOX but induced less toxicity-induced weight loss compared to that of free DOX.

### 3.8. DOX-Induced Cardiotoxicity Is Avoided with Phytogenic TiO_2_ NPs

**Figure 10:**
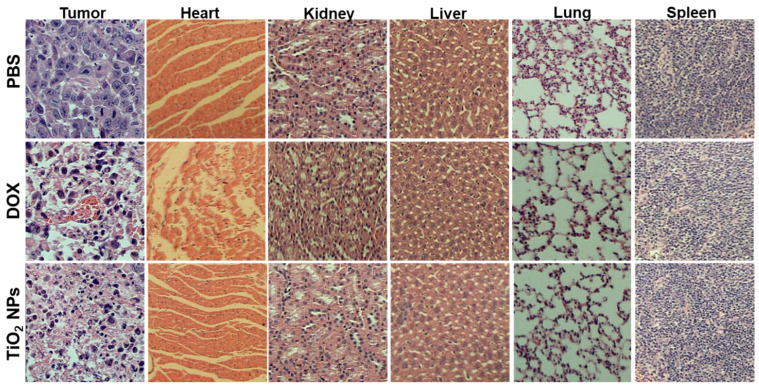
Histopathological analysis (at day 21) of the induced-breast-tumor tissue and major surrounding organs of breast-tumor-bearing BALB/c mice after treatment with PBS (1×, pH 7.4; 20 mL/kg/day), free DOX (5 mg/kg/day), or TiO_2_ NPs (5 mg/kg/day).

Subsequently, the apoptotic characteristics were analyzed (at day 21) histologically from H&E-stained sections of the induced murine breast tumor and other major surrounded organs (i.e., heart, kidney, liver, lung, and spleen) (Figure 10).

The H&E-stained sections obtained from the induced breast tumor displayed a primary cellular structure of tumor cells, evenly scattered in the PBS group used as NC. The apoptosis signs, such as cell shrinkage, chromatin condensation (pyknosis), eosinophilic cytoplasm and evenly dense nuclei, were observed in the tumoral breast tissue of TiO_2_ NPs- and DOX-treated mouse groups. In addition, a remarkable decrease in vascular density was also seen in the breast tumoral tissue of TiO_2_ NPs-treated mouse group, which strongly suggested inefficient delivery of essential nutrients and oxygen to the growing tumor cells.

Although no apparent histopathological changes were detected in the kidney, liver, lung, and spleen after DOX or TiO_2_ NPs treatment in tumor-bearing mice compared to that of PBS-treated tumor-bearing mice, the heart-stained sections obtained from DOX-treated mice displayed clear neutrophils infiltration and irregular vasculature compared to that of TiO_2_ NPs- and PBS-treated tumor-bearing mice.

Thus, our data demonstrated that the greenly synthesized TiO_2_ NPs do not induce cardiotoxicity, a common feature of DOX treatment [66,67].

## 4. Conclusions and Perspectives

In the current study, TiO_2_ NPs were prepared by a simple, fast, efficient, cost-effective, and green method using butanolic leaf extract of *Z. armatum*, a plant endemic in Pakistan and China. The phytogenic NPs were physically characterized mainly to get information about their bonding system, surface topology, nature, particle size and particle geometry. The successful fabrication of TiO_2_ NPs was confirmed by UV and FTIR analyses. Further, the XRD pattern confirmed their tetragonal anatase crystalline geometry with a small core particle size of 16.2 ± 2 nm, also observable by TEM.

Interestingly, potent DOX-like breast anticancer activity of *Z. armatum*-derived TiO_2_ NPs was demonstrated both ex vivo (in 4T1 mammary carcinoma cells) and in vivo (4T1-induced breast carcinoma in BALB/c mice). Their mode of anti-tumoral action was shown to be more likely mediated by ROS generation causing LPO. Importantly, the TiO_2_ NPs were found suitable for IV administration, and displayed significantly less cardiotoxicity and body weight loss compared to that of DOX.

Taken together, this original research study pointed out the efficiency, the safe applicability, and thus the superiority of using *Z. armatum*-derived TiO_2_ NPs over free DOX, a potent chemotherapeutic drug commonly used to treat breast cancer worldwide. This concept and a rational use of green smart mesoporous TiO_2_ NPs may be implemented in the pharmaceutical industry to develop more effective therapeutic regimen for breast cancer.

## 5. Significance Statement

The present study reports the green and cost-effective synthesis of small, spherical TiO_2_ NPs of crystalline nature, by using *Z. armatum* (timer) leaf extract as a unique reducing agent, and butanol as a templating agent. The usefulness of this simple approach for the production of mesoporous material with tunable sizes have advantages over existing routes. This study (i) reports a new route to biosynthesize TiO_2_ NPs; (ii) demonstrates that TiO_2_ NPs are as efficient as DOX toward breast carcinoma ex vivo and in vivo; (iii) reveals a new paradigm shift that TiO_2_ NPs exert an inherent anticancer activity, more likely by a molecular mechanism involving ROS-induced cell death; (iv) demonstrates that *Z. armatum*-derived TiO_2_ NPs are not cardiotoxic and do not alter the body weight, making them a safer agent compared to DOX; (v) shall help researchers to shortlist metallic NPs exerting such safe effects as well as cytotoxic potential towards cancer cells and tumors in order to develop smart/advanced chemotherapeutic formulations.

## Data Availability

Data sharing not applicable.

## Abbreviations

DLS	Dynamic Light Scattering
DOX	Doxorubicin
FACS	Fluorescence-activated cell sorting
FTIR	Fourier Transform-Infrared (spectroscopy)
HR	Hemolysis Rate
LPO	Lipid Peroxidation
MTT	3-(4,5-Dimethylthiazol-2-yl)-2,5-diphenyltetrazolium bromide
NPs	Nanoparticles
PBS	Phosphate-Buffered Saline
PI	Propidium Iodide
PS(D)	Particle Size (Distribution)
RBCs	Red Blood Cells
ROS	Reactive Oxygen Species
SEM	Scanning Electron Microscopy
TEM	Transmission Electron Microscopy
TiO_2_	Titanium dioxide
UV-Vis	Ultraviolet-Visible
XRD	X-Ray powder Diffraction
*Z. armatum*	*Zanthoxylum armatum*
