# Correction: Iqbal et al. Breast Cancer Inhibition by Biosynthesized Titanium Dioxide Nanoparticles Is Comparable to Free Doxorubicin but Appeared Safer in BALB/c Mice. *Materials* 2021, *14*, 3155

**DOI:** 10.3390/ma19020420

**Published:** 2026-01-21

**Authors:** Haroon Iqbal, Anam Razzaq, Bushra Uzair, Noor Ul Ain, Shamaila Sajjad, Norah Ayidh Althobaiti, Aishah Eid Albalawi, Bouzid Menaa, Muhammad Haroon, Muslim Khan, Naveed Ullah Khan, Farid Menaa

**Affiliations:** 1 College of Pharmaceutical Science, Soochow University, Suzhou 215123, China; harooniqbal415@hotmail.com (H.I.); anamrazzaq.ajk@gmail.com (A.R.); noorulain22@yahoo.com (N.U.A.); naveedkhan1676@hotmail.com (N.U.K.); 2 Department of Biological Sciences, International Islamic University, Islamabad 44000, Pakistan; bushra.uzair@iiu.edu.pk (B.U.); shamaila.sajjad@iiu.edu.pk (S.S.); 3 Department of Biology, Faculty of Science and Humanities, Shaqra University, Al-Quwayiyah 11961, Saudi Arabia; nalthobaiti@su.edu.sa; 4 Department of Biology, Faculty of Sciences, University of Tabuk, Tabuk 47731, Saudi Arabia; ae.albalawi@ut.edu.sa; 5 Department of Oncology and Nanomedicine, California Innovations Corporation, San Diego, CA 92037, USA; bouzid.menaa@gmail.com; 6 Faculty of Pharmacy, Gomal University, Dera Ismail Khan 29050, Pakistan; haroon.pharma1717@gmail.com; 7 Department of Chemistry, Kohat University of Science and Technology, Kohat 26000, Pakistan; dr.muslim@kust.edu.pk

In the original publication [1], there were overlaps in Figure 10 as published. The corrected Figure 10 appears below. The authors state that the scientific conclusions are unaffected. This correction was approved by the Academic Editor. The original publication has also been updated.

## Figures and Tables

**Figure 10:**
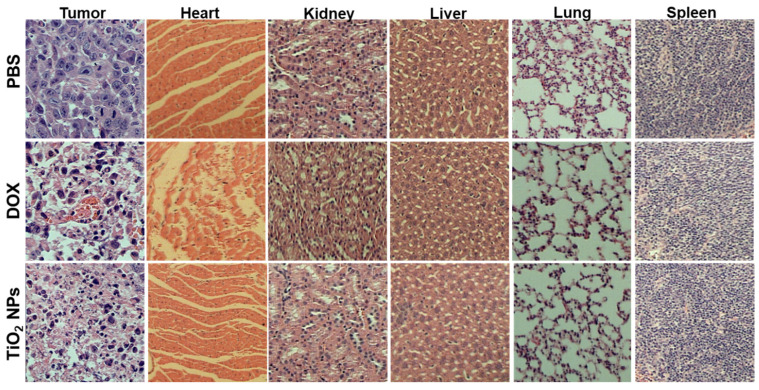
Histopathological analysis (at day 21) of the induced-breast-tumor tissue and major surrounding organs of breast-tumor-bearing BALB/c mice after treatment with PBS (1×, pH 7.4; 20 mL/kg/day), free DOX (5 mg/kg/day), or TiO_2_ NPs (5 mg/kg/day).

## References

[1] Iqbal H., Razzaq A., Uzair B., Ain N.U., Sajjad S., Althobaiti N.A., Albalawi A.E., Menaa B., Haroon M., Khan M. (2021). Breast Cancer Inhibition by Biosynthesized Titanium Dioxide Nanoparticles Is Comparable to Free Doxorubicin but Appeared Safer in BALB/c Mice. Materials.

